# Error-corrected sequencing strategies enable comprehensive detection of leukemic mutations relevant for diagnosis and minimal residual disease monitoring

**DOI:** 10.1186/s12920-020-0671-8

**Published:** 2020-03-04

**Authors:** Erin L. Crowgey, Nitin Mahajan, Wing Hing Wong, Anilkumar Gopalakrishnapillai, Sonali P. Barwe, E. Anders Kolb, Todd E. Druley

**Affiliations:** 1 Biomedical Research Department, Nemours / A.I. DuPont Children’s Hospital, Wilmington, DE USA; 2 Department of Pediatrics, Washington University School of Medicine, 660 South Euclid Avenue, Campus Box 8116, St. Louis, MO 63110 USA; 3 Edison Family Center for Genome Sciences and Systems Biology, Washington University School of Medicine, 4515 McKinley Avenue, Campus Box 8510, St. Louis, MO 63108 USA; 4 Nemours Center for Cancer and Blood Disorders, Nemours/A.I. duPont Hospital for Children, Wilmington, USA

**Keywords:** Error-corrected sequencing, Minimal residual disease, Next generation sequencing, Pediatric leukemia, Computational biology

## Abstract

**Background:**

Pediatric leukemias have a diverse genomic landscape associated with complex structural variants, including gene fusions, insertions and deletions, and single nucleotide variants. Routine karyotype and fluorescence in situ hybridization (FISH) techniques lack sensitivity for smaller genomic alternations. Next-generation sequencing (NGS) assays are being increasingly utilized for assessment of these various lesions. However, standard NGS lacks quantitative sensitivity for minimal residual disease (MRD) surveillance due to an inherently high error rate.

**Methods:**

Primary bone marrow samples from pediatric leukemia (*n* = 32) and adult leukemia subjects (*n* = 5), cell line MV4–11, and an umbilical cord sample were utilized for this study. Samples were sequenced using molecular barcoding with targeted DNA and RNA library enrichment techniques based on anchored multiplexed PCR (AMP®) technology, amplicon based error-corrected sequencing (ECS) or a human cancer transcriptome assay. Computational analyses were performed to quantitatively assess limit of detection (LOD) for various DNA and RNA lesions, which could be systematically used for MRD assays.

**Results:**

Matched leukemia patient samples were analyzed at three time points; diagnosis, end of induction (EOI), and relapse. Similar to flow cytometry for ALL MRD, the LOD for point mutations by these sequencing strategies was ≥0.001. For DNA structural variants, FLT3 internal tandem duplication (ITD) positive cell line and patient samples showed a LOD of ≥0.001 in addition to previously unknown copy number losses in leukemia genes. ECS in RNA identified multiple novel gene fusions, including a *SPANT-ABL* gene fusion in an ALL patient, which could have been used to alter therapy. Collectively, ECS for RNA demonstrated a quantitative and complex landscape of RNA molecules with 12% of the molecules representing gene fusions, 12% exon duplications, 8% exon deletions, and 68% with retained introns. Droplet digital PCR validation of ECS-RNA confirmed results to single mRNA molecule quantities.

**Conclusions:**

Collectively, these assays enable a highly sensitive, comprehensive, and simultaneous analysis of various clonal leukemic mutations, which can be tracked across disease states (diagnosis, EOI, and relapse) with a high degree of sensitivity. The approaches and results presented here highlight the ability to use NGS for MRD tracking.

## Background

Genetic characterization of leukemias is a critical component of the clinical evaluation, risk stratification, and therapeutic strategy [1]. Chromosomal rearrangements generating gene fusions and complex structural variants (StVs) are more common in childhood than in adult leukemias (M. J [2].). Recently, genomic data analysis of cancer patients has highlighted the significance of these genomic rearrangements and single nucleotide variants (SNVs), causing an increase in the use of site-specific qPCR, RT-qPCR or gene expression profiling for diagnostic characterization (M. J [3].). However, leukemias are heterogenous at the genomic level, with many rare variants driving clinically indistinguishable disease. Single variant assays require a priori knowledge of the mutations present, thus limiting the scalability of such an approach.

Minimal residual disease (MRD; more aptly named Measurable Residual Disease) is the detection of residual leukemia following therapy, most commonly by flow cytometry. MRD, as measured by multiparameter flow cytometry is perhaps one of the most important predictors of outcome in children with acute myelogenous leukemia (AML) [4]. For pediatric acute lymphoblastic leukemia (ALL) flow cytometry data, with a limit of detection of 0.0001 for clonal B- or T-cell surface receptors, the “leukemia-associated immunophenotype” (LAIP), has been shown to correlate with significantly worse disease-free and overall survival (M. J [3].; M. J [2].).

In contrast, because myeloid cells do not harbor a single clonal surface marker, multi-parameter flow cytometry (MPFC) for a profile of surface immunophenotypes displaying a “different from normal” cell population is the current gold standard for MRD in AML with a limit of detection of approximately 0.001 [4]. However, surface immunophenotypes often change during AML therapy [5]. Moreover, MPFC does not enable identification of genomic lesions that are associated with treatment options and risk stratification.

Standard NGS platforms have systematic error rates of approximately 0.005–0.02 (A. L. [6]; A. L. [7]), depending on the platform and analytic strategy. Thus, NGS strategies are limited in their ability to detect variants at allele frequencies below the error rate of the platform, plus the sequencing depth requirements for bulk sequencing to achieve these limits of detection are cost prohibited. Error corrected sequencing (ECS) mitigates systematic errors via the incorporation of a unique molecular index (UMI) to each molecule captured (A. L. [6]) allowing for errors to be discarded and very rare mutations to be clearly detected in heterogeneous nucleic acid samples. Furthermore, by coupling UMIs with anchored multiplexed PCR (AMP) technology, the robust identification of StVs in DNA, including copy number variations, or RNA, including cryptic gene fusions, is enabled.

Adding UMIs across an entire genome or transcriptome is cost prohibitive, and there is no “one size fits all” strategy for detecting and tracking the wide variety of mutation in any given cancer. In this study, our goals were to demonstrate various quantitative strategies for comprehensive detection of complex, low frequency mutations from a collection of leukemia samples with known diagnostic results and outcomes. The data presented demonstrate the ability to create NGS strategies capable of reaching limits of detection appropriate for MRD. Furthermore, the results highlight the robust ability of ECS to quantitatively assess the complex mutational landscape of leukemias, which will facilitate precision therapeutic selection and ultimately reduced morbidity and improved survival.

## Methods

### Samples and consent

All samples used in this study were collected from human peripheral blood or bone marrow. Specimens were collected for biobanking and subsequent de-identified biomedical research at Nemours following written informed consent (Human Research Protection Office (HRPO) IRB# 349465), including parental permission forms (child/adolescent assent was obtained for ages 7–17 years). The Nemours samples used in this study were deemed non-human research and approved by the Nemours / A.I. duPont Children’s Hospital (HRPO IRB# 267207). Patients enrolled on the Children’s Oncology Group (COG) AAML1031 phase III pediatric de novo AML study (NCT00372593) had the option to consent/assent for correlative biomedical research when enrolling for the study. All available COG samples were de-identified and personal health information (PHI) was not available to study team. Adult AML specimens are banked at Washington University for biomedical research under Human Research Protection Office (HRPO) IRB# 201011766. This study was approved as by the Washington University HRPO IRB# 201511125 entitled “Error-corrected sequencing for minimal residual disease in AML”.

Samples from Washington University included an umbilical cord sample and adult (*n* = 5) de novo AML samples with known *FLT3-ITD* lesions. COG samples were collected from pediatric de novo AML patients enrolled on the COG Phase III prospective study, AAML1031. In total we sequenced 4 unique subjects, with each subject having 2 or 3 timepoints collected (diagnosis, end of induction (EOI), relapse). The Nemours samples consisted of six AML subjects, one acute promyelocytic leukemia (APL) subject, 17 preB-cell ALL subjects and three T-cell ALL subjects (Table 1). Demographic data for these samples are summarized in Additional file 5: Table S1. The sample distribution matches with the incidence rate of pediatric AML, APL, B-ALL and T-ALL reported in US population, respectively.

**Table 1:**
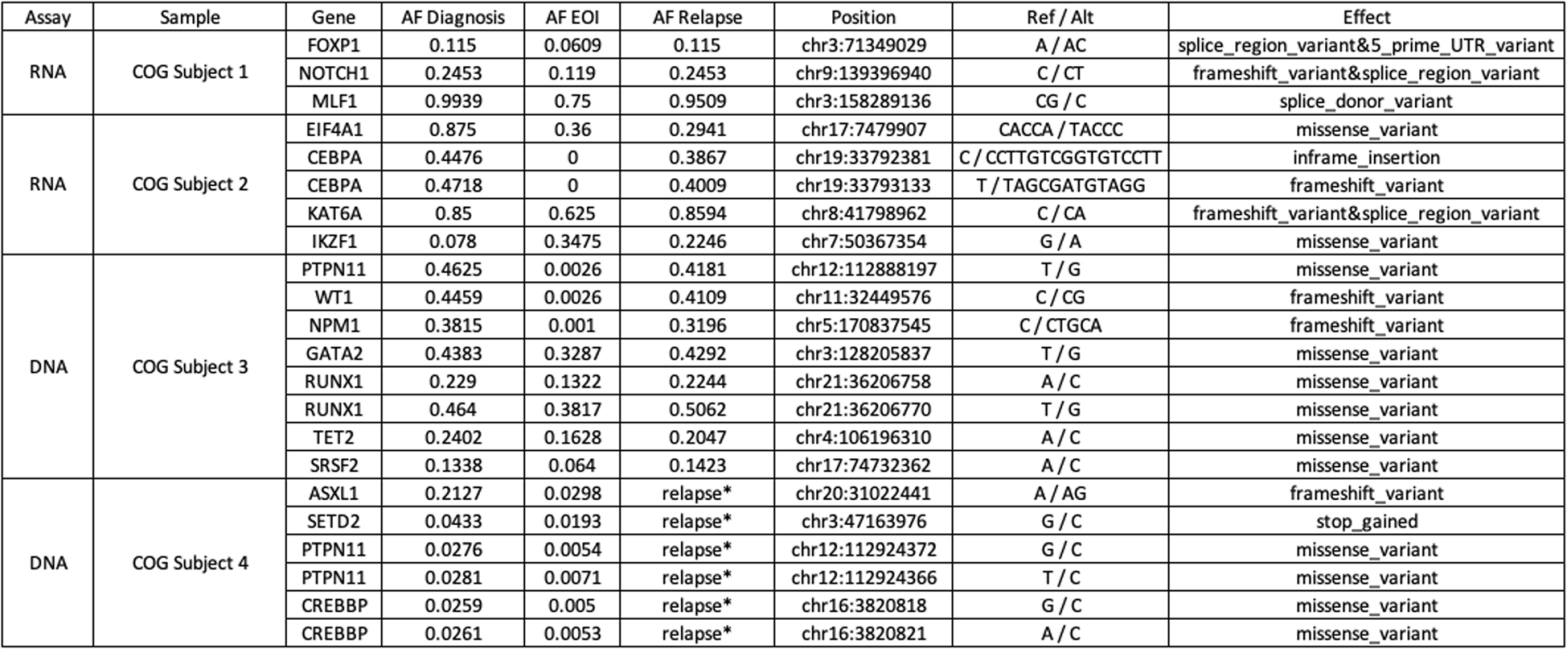
Summary of variants and allele frequencies in longitudinal samples

### Nucleic acid extraction

Nucleic acid was extracted from each sample in the Druley laboratory at Washington University. DNA was extracted using the Qiagen (Germantown, Maryland, USA) DNeasy Blood & Tissue Kit (catalog #: 69504) using the manufacturer’s protocol. RNA was extracted using the Qiagen RNeasy Plus Mini Kit (catalog #: 74134). Nucleic acid quantity and quality was then assessed using the Agilent (Santa Clara, California USA) TapeStation 4200 following the manufacturer’s protocol using the High Sensitivity D1000 Screen Tape (catalog #: 5067–5584) for DNA or the High Sensitivity RNA Screen Tape (catalog #: 5067–5579) for RNA.

### DNA-ECS library preparation

To optimize detection of structural and copy number variants in DNA and RNA in genes more closely affiliated with pediatric leukemia, we prepared DNA-ECS libraries using a customized ArcherDx (Boulder, Colorado USA) VariantPlex kit based on published data from the Therapeutically Applicable Research to Generate Effective Treatments (TARGET) project conducted by COG [8]. The custom assay consists of 1395 primer pairs and required 250 ng of DNA for input. Primer amplification uniformity was > 95% for the all assays.

### RNA-ECS library preparation

There were two different RNA-ECS library preparation methods used in this study. First, for absolute quantification of transcript copy number in 416 human cancer related genes, we adapted the Qiagen Human Cancer Transcriptome kit (catalog #: RHS-003Z) to be compatible with our UMI-aware bioinformatics, similar to our published DNA method (A.L. [7]). Total RNA was extracted using RNeasy Mini Kit (Qiagen, Inc), and cDNA was made from 50 ng of RNA using the QIAseq kit. Each cDNA molecule from the region of interest was then tagged with an UMI prior to PCR amplification. Libraries were made using the QIAseq kit following the manufacturer’s protocol. For each sample, a technical replicate was also made. Second, for quantitative characterization of structural variation in mRNA (fusions, aberrant splice isoforms, retained introns), the ArcherDX FusionPlex HemeV2 Kit (catalog #: AB0012) was utilized per manufacturer’s protocols.

### Bioinformatics

The data analysis for the ArcherDx library preparation followed the following steps: read quality cleaning, error correction, genome alignment, and variant detection and annotation. Fastq files were analyzed via FASTQC for quality, and trimmed based on adaptor sequences and quality. Trimmed reads were aligned to hg19 using bwa mem, bowtie2, and mummer3, whereas de novo reference assembly was conducted by velvet and AMRA (Additional file 1: Figure S1). SNVs and short InDels (≤20 bp) were detected from the genomic alignments by freeBayes and Lofreq, whereas large InDels (> 20 bp) were detected via custom de novo assembly. Variants were filtered based on depth of error-corrected sequencing bins, minimum of 3, that supported the call. All regions in which variants were called required a total read depth > 100X, and a minimal base quality score (phred) of 20 was applied. The ExAC database was used to annotate common variants. Alignment files were processed through several algorithms for variant detection. Data analysis was conducted using an Amazon Web Service that hosted the ArcherDx Analysis platform (v5.1.3).

### Copy number variation analysis

CNV filtering was based on the following: 1) a *p*-value calculation, based on a two-tailed Wilcoxon rank sum test, with a null hypothesis that the median value of the copy number called for each probe of the given target was equal to the median value of the copy number called for all primers identified as being in the baseline, across all samples analyzed; 2) standard deviation of the called copy number for all probes of the target; 3) total number of adjacent gene-specific primers (GSPs) supporting the CNV event; and 4) variants deemed likely to be a chemistry or sequencing artifact were filtered out.

### Variant allele determination for complex variants

The variant allele frequency (VAF) for a *FLT3* internal tandem duplication (*FLT3-ITD*) is calculated by comparing reads supporting the wild type (WT) *FLT3* junction in that region, to the reads supporting a novel junction. To optimize detection of variably sized duplications/deletions, the small ITD (< 20 bp) are detected via forced reference alignment, and the larger (> 20 bp) ITD are detected via de novo assembly, as highlighted in Additional file 1: Figure S1. It is essential to consider the following characteristics of the assay: read depths, sample complexity, depth of coverage, breadth of coverage, molecular bin sizes, and power analysis to detect low allelic variants. We analyzed the following: (1) coverage per targeted base at different read depths, (2) coverage versus error correctable coverage for all targeted bases – 35 million and full depth, (3) percentage of targeted bases covered at 35 M reads per sample, (4) percent of targeted bases covered at various read depths (5) statistical power to call low allelic variants based on background noise at that position and cohort level sequencing metrics (6) summary of error correctable coverage and sensitivity for all targeted bases at individual sample level.

For RNA-ECS, raw sequencing reads sharing the same UMIs were aligned to each other to form read families as per DNA-ECS analysis. A minimum of three reads per UMI was required for the downstream process of de-duplication and error-correction. A consensus read was then made for each read family and aligned to hg19.

## Results

### Application of ECS for MRD

**Fig. 1:**
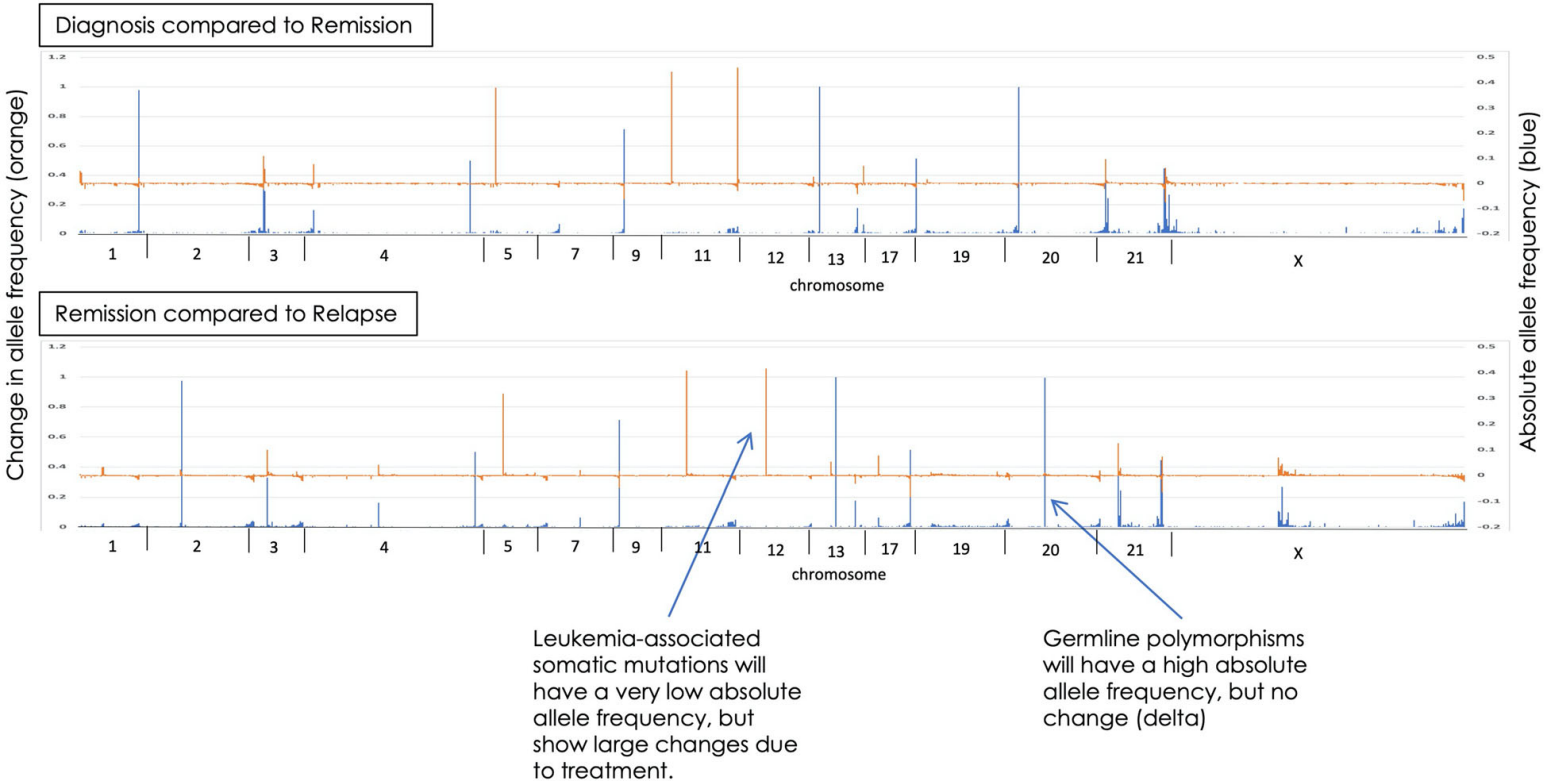
Variant tracking at diagnosis, end of induction (EOI), and relapse in a single pediatric AML subject across multiple regions in the genome. The top panel represents the analysis between the diagnostic and end of induction sample. Left axis and blue lines represent the variant allele frequency (VAF), and the orange lines and right x-axis represent the delta of VAF between the diagnostic and end of induction sample. The bottom panel of the Figure represents the analysis between endo of induction and relapse with the same x- and y- axis as the top panel

**Fig. 2:**
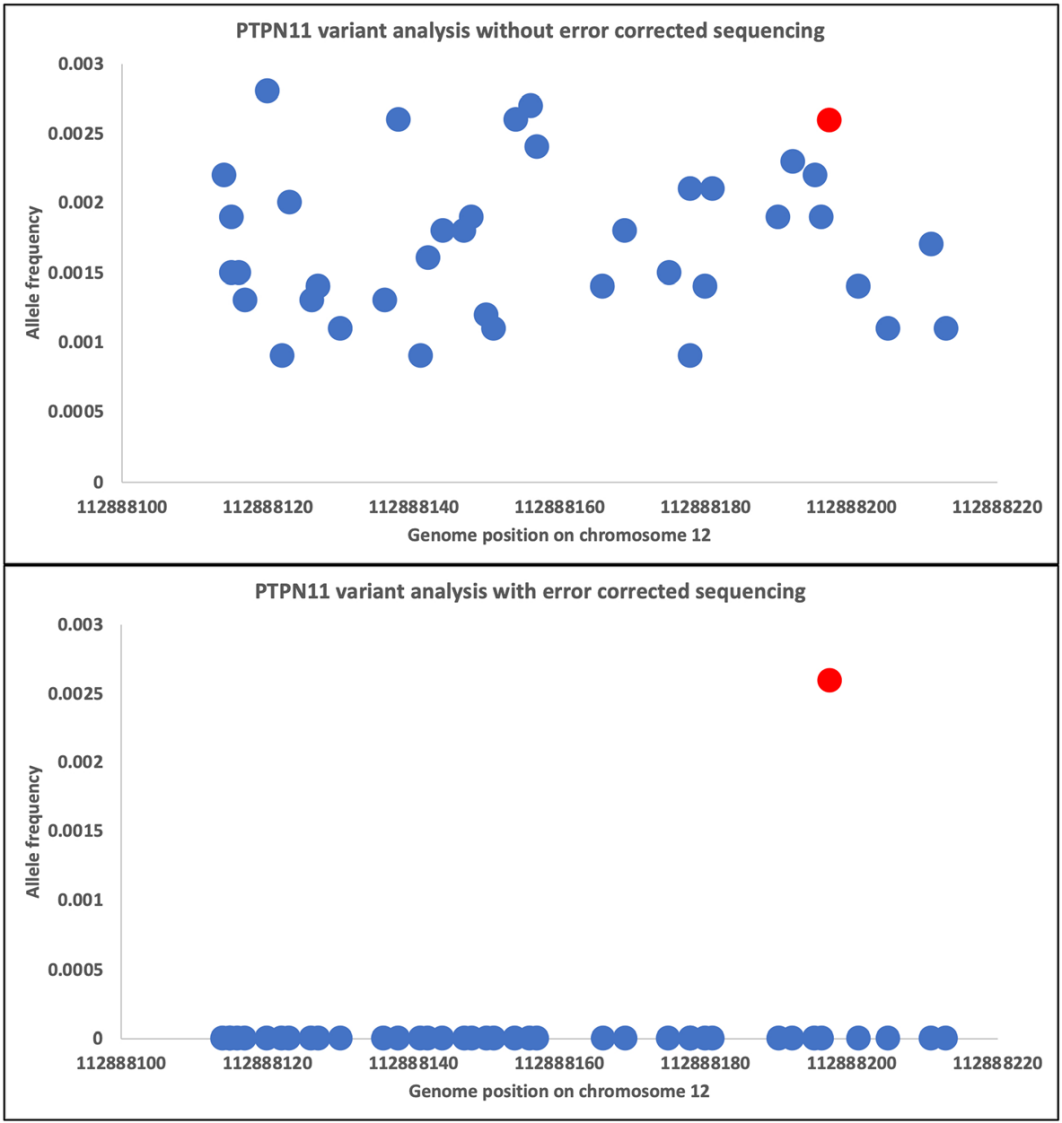
Error-corrected sequencing noise reduction for low allelic variants. Top panel represents all variants (blue dots) identified in the genomic region of a known somatic mutation located in *PTPN11* (red dot). Bottom panel demonstrates noise reduction with the application of error-corrected sequencing

To assess the limits of detection (LOD) in a clinically meaningful scenario, we retrospectively applied our custom DNA- and RNA-ECS assays to longitudinal pediatric leukemia samples collected from four unique subjects. Each subject had 2 or 3 timepoints collected (diagnosis, EOI, relapse). COG subject 3 had all time points analyzed, diagnosis, EOI, and relapse, and the results are demonstrated in Fig. 1. The top panel in Fig. 1 represents the analysis between diagnosis and EOI, with the left axis and blue lines representing VAF across the genome, and the orange lines and right x-axis representing the delta, or change, in VAF between diagnosis and EOI. The data demonstrate the detection of noise and disease relevant variations. The bottom panel in Fig. 1 represents the analysis between EOI and relapse. Using the EOI sample to help annotate germline variants, in total 40 germline mutations were detected, 5 mosaic like variants, and 4 somatic variants were tracked across all 3 samples: *PTPN11*, *WT1*, *NPM1*, and *FLT3-ITD*. Each of these variants were detected at EOI using error-corrected sequencing reads, with VAF ranging from 0.001–0.0026, which are frequencies below the LOD for NGS without molecular indexing and consistent with leukemic cell LOD by flow cytometry for MRD. ECS provides noise reduction that permits detection of variants with low allele frequencies that are below the limit of detection by bulk sequencing strategies (WES data from TARGET). Figure 2 highlights the ECS background noise reduction (blue dots) for the *PTPN11* variant (red dot) identified at a low allelic frequency after induction therapy.

Table 1 summarizes the variants identified in the 4 subjects analyzed. Of interest, COG subject 2 had an IKZF1 mutation at low allelic frequency at time of diagnosis (0.078), which increased at both the EOI (0.3475) and relapse state (0.2246). COG subject 1 had a *NOTCH1* and *FOXP1* splice region variants that were detectable at EOI (Table 2). COG subject 4 relapsed, but the sample was not available for analysis and only the diagnostic and EOI samples have been analyzed, which indicated several potential variants of interest for disease tracking (Table 1).

**Table 2:**
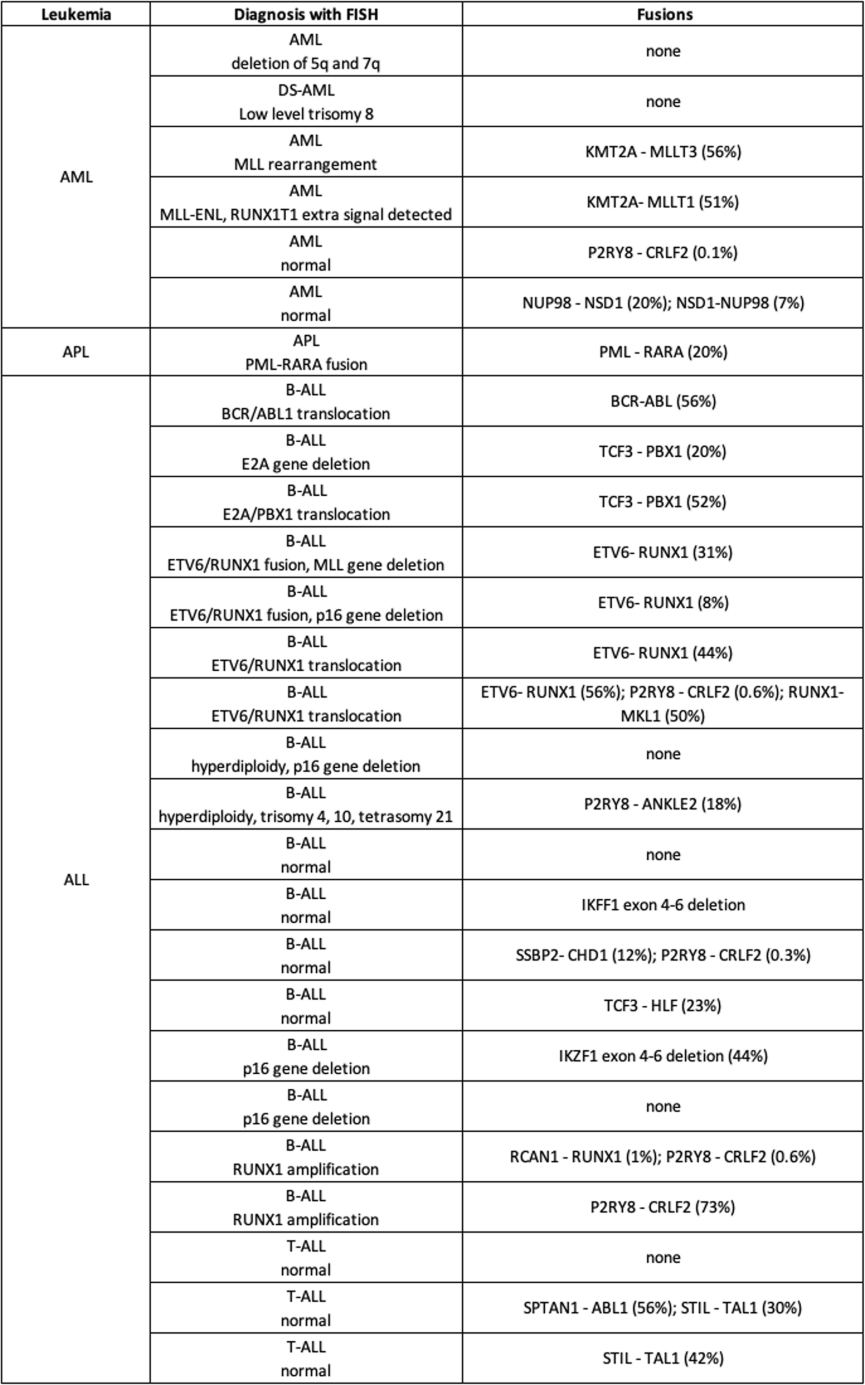
Comparison between gene rearrangements detected by diagnostic FISH analysis and by targeted error-corrected sequencing of pediatric primary bone marrow specimens

### Limit of detection (LOD) for FLT3-ITD

**Fig. 3:**
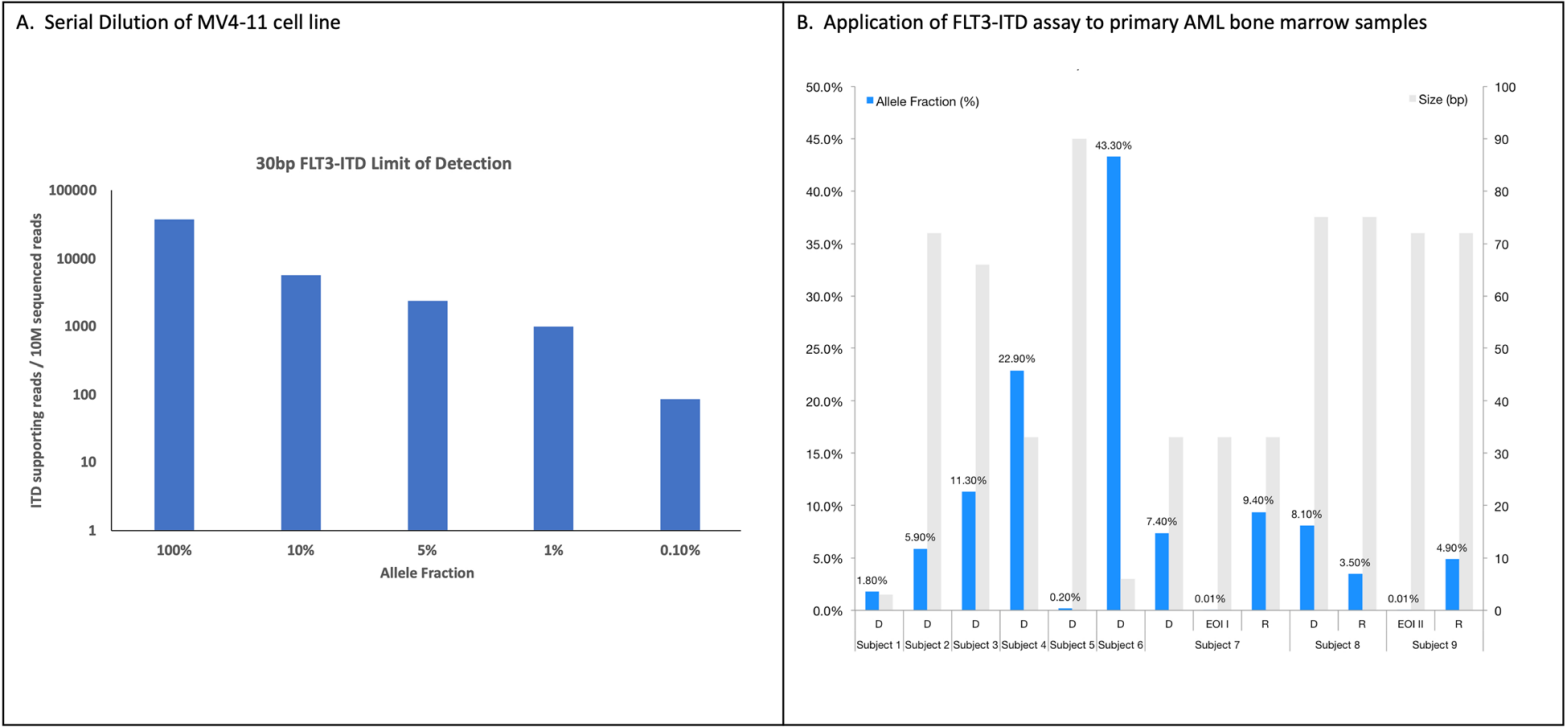
Development of a specific and sensitive NGS panel for *FLT3-ITD* detection and disease monitoring. **a** Serial dilution results for MV4–11 cell line (30 bp ITD in *FLT3*). The x-axis is allele fraction and y-axis is the number of ITD supporting reads / 10 M sequencing reads. **b** Nine leukemia samples were analyzed with varying ITD sizes (right y-axis, grey bars) and allele fraction (left y-axis, blue bars). Subjects are across the x-axis, with D = diagnosis, EOI = end of induction, and R = relapse sample

To analyze the limit of detection (LOD) for complex variants, we focused on *FLT3-ITD* in DNA (Additional file 6: Table S2). To determine the LOD, DNA from a human leukemia cell line (MV4–11) with a 30 bp *FLT3-ITD* was serially diluted into human genomic DNA followed by DNA-ECS. The 30 bp *FLT3-ITD* was detected in all samples at expected frequencies (Fig. 3a). Given the variable size of *FLT3-ITDs*, from a few bases to hundreds, we utilized primary bone marrow samples from AML subjects (*n* = 9) with known *FLT3-ITD*s to establish a range of allele detection frequencies and sizes. DNA-ECS detected all of the ITDs across different sizes (range 3–90 bp) and frequencies (0.0001–0.43) as shown in Fig. 3b. These data further demonstrate the ability to detect *FLT3-ITD* mutations with molecular diagnostics at a LOD appropriate for MRD detection.

Additionally, by using the primer locations for all target genes, ECS coupled with AMP technology enables detection of copy number gains or losses. The computational pipeline identified a loss of the *CBL* locus (E3 ubiquitin-protein ligase CBL) in one child (Additional file 3: Figure S3). The deletion is smaller than would be detected by cytogenetics or SNP array, but large enough to cause loss of PCR amplification that could be interpreted as a false negative for variation if only the wild type allele is amplified. Overall, these results provide better resolution and increased breadth of detectable lesions to the standard assays, as the position and sequence, along with accurate quantitation, is accomplished in a single assay.

### Digital quantification of RNA transcripts

By incorporating UMIs to the targeted enrichment of cDNA molecules prior to amplification and sequencing, the ECS-RNA eliminates the issue of duplication bias, thus enabling digital, rather than relative (such as transcriptome sequencing), quantification of gene expression. As a proof of principle, we surveyed for gene expression patterns in a healthy umbilical cord blood and a pediatric AML remission sample using a commercial product that targets 416 cancer-associated genes. RNA-ECS can quantitatively detect mRNA copy numbers as low as < 10 copies, which was validated by droplet digital PCR (Additional file 4: Figure S4).

### Characterization of diagnostic leukemia samples using error-correct sequencing techniques

To demonstrate the utility of our ECS capture technique for the detection of novel complex RNA variants, 27 primary pediatric diagnostic samples were analyzed (*n* = 6 AML; *n* = 1 APL, *n* = 17 preB-ALL, *n* = 3 T-ALL; Additional file 5: Table S1 demographics). Nine out of the 27 primary bone marrow samples contained gene fusions detected by routinely tested FISH probes for diagnostic purposes consisting of *ETV6-RUNX1* (*n* = 4 subjects), *BCR-ABL1* (n = 1 subject), *TCF3-PBX1* (n = 1 subject), *PML-RARA* (n = 1 subject) and *KMT2A* rearrangements (*n* = 2 subjects). ECS-RNA not only confirmed these gene fusions but also detected previously undetected cryptic gene fusions in 10 subjects that were negative for chromosomal rearrangements via FISH. Some of these cryptic gene fusions, including *NUP98-NSD1*, *P2RY8-CRLF2* and *TCF3-HLF*, are recurrently seen in pediatric leukemias and their prognostic significance has been demonstrated [9–11]. Other identified gene fusions, such as STIL-TAL1, have an unclear role in T-ALL biology (A.L. [7]). Additionally, we identified novel in-frame gene fusions - *SPTAN1-ABL1*, *SSBP2-CHD1*, *RUNX1-MKL1*, *RCAN1-RUNX1*, and *P2RY8-ANKLE2* (Table 2), which were confirmed by Sanger sequencing. One subject with an *ETV6-RUNX1* fusion detected by FISH, showed two additional cryptic gene fusions by RNA-ECS (Table 2).

The remaining 6 subjects did not harbor detectable gene fusions by RNA sequencing. Two preB-ALL samples showed deletion of *IKZF1* exons 4–6 (Additional file 7: Table S3); which have been reported in 9% of B-ALL patients [12] and results in expression of the dominant negative form of the transcription factor IKAROS lacking DNA binding zinc finger motifs [13]. Four unique and previously unidentified gene fusions via karyotyping data at diagnosis were identified in primary AML samples, *KMT2A-MLLT1*, *KMT2A-MLLT3*, *NUP98-NSD1* and the reciprocal *NSD1-NUP98*, and 5 exon duplication/deletions were identified in *CEBPA* and *IRF4* (Table 2 and Additional file 7: Table S3). These results demonstrate the diversity of prognostic or therapeutically relevant somatic mutations inherent in leukemias, which are often undetectable by gold standard methods.

**Fig. 4:**
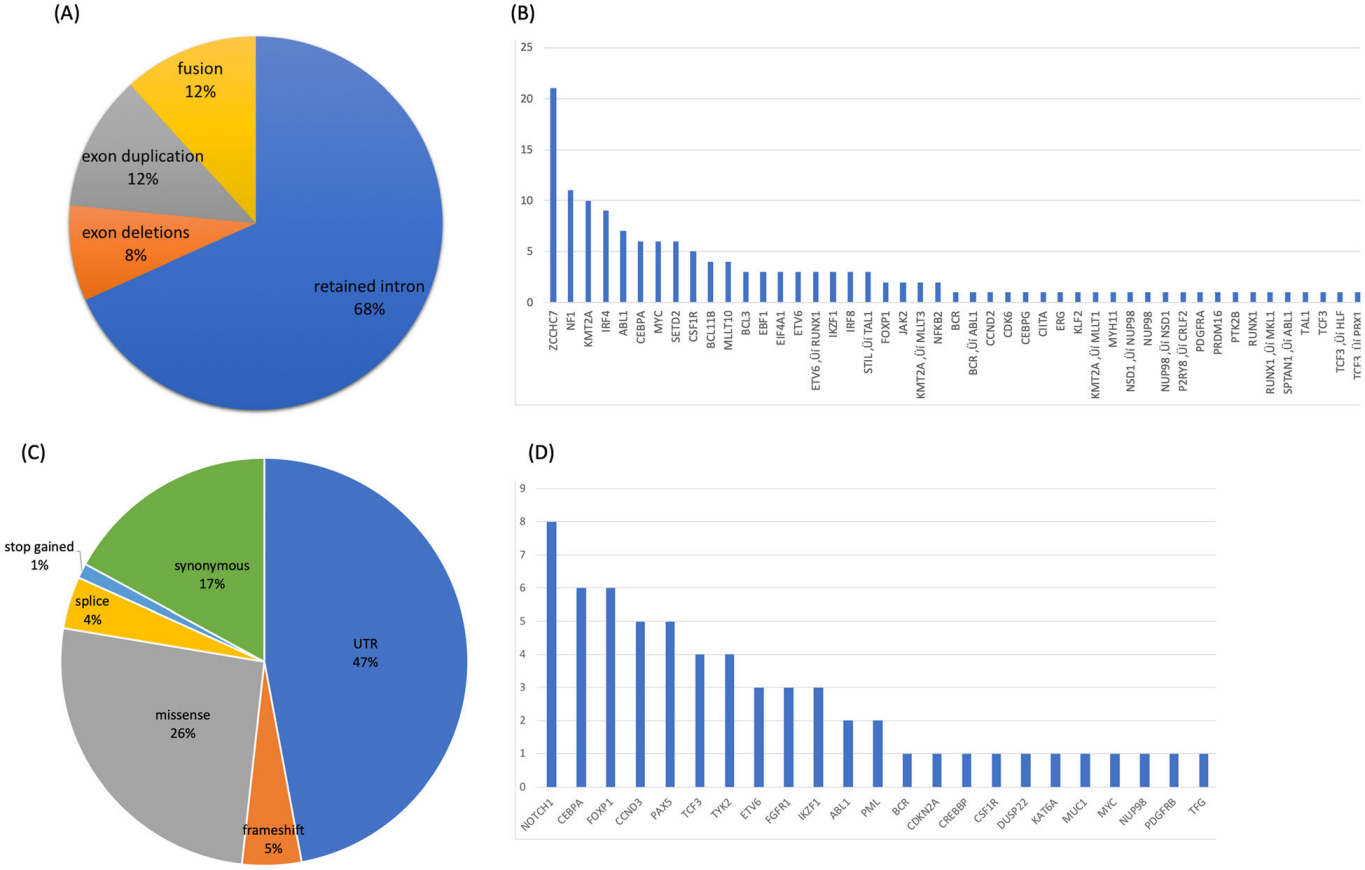
Summary of RNA-ECS results for pediatric leukemia diagnostic samples. **a** Distribution of allelic specific single nucleotide variants and gene counts. Pie chart represents the distribution across all leukemia samples. **b** The bar graph represents the counts per gene. **c** Distribution of RNA StVs and gene counts. Pie chart represents the distribution across all leukemia samples for SNVs. **d** The bar graph represents the counts per gene

**Fig. 5:**
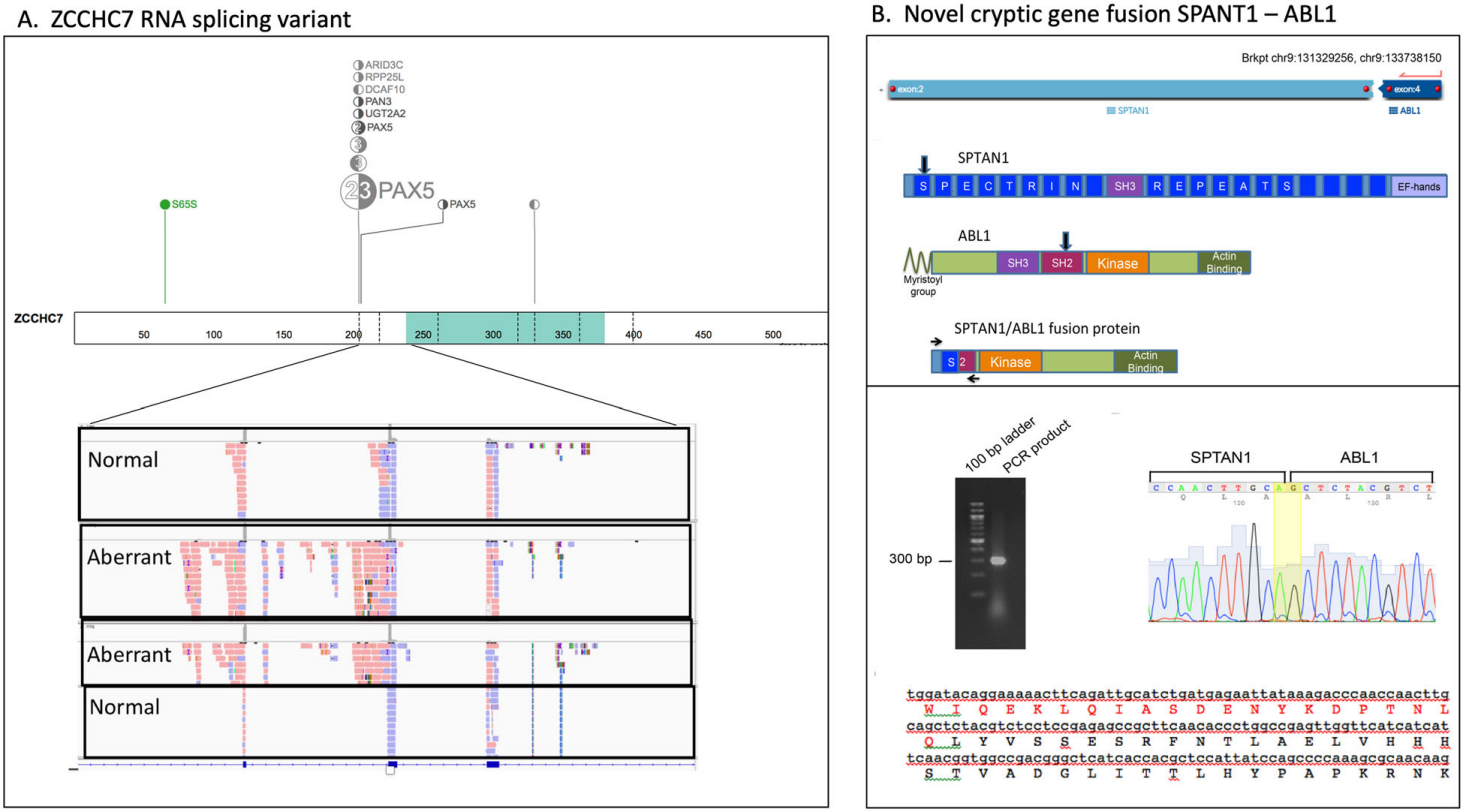
Novel RNA variants identified at time of diagnosis. **a** An aberrant RNA molecule in *ZCCHC7* was identified in several of the B-ALL samples. **b** Novel cryptic gene fusion in *SPTAN1-ABL1* was identified in a T-ALL subject and confirmed via Sanger sequencing. ExPASy translation of the fusion product sequence revealed the in-frame fusion of *SPTAN1* (amino acids in red) and *ABL1* (amino acids in black)

In total, 143 RNA StVs were identified (Fig. 4a and Additional file 7: Table S3). All samples showed alternative exon usage and domain duplications/deletions for several known oncogenes along with novel mutations. We observed exon duplications in *MYC* (AF range 0.13–0.88), *BCL11B* (AF range 0.12–.22), *CEBPA* (AF range 0.10–0.79), and *ZCCHC7* (AF range 0.11–0.83), while exon deletions were noted in *IRF4* (AF range 0.12–0.55), *IKZF1* (AF range 0.40–0.44) and *SETD2* (AF range 0.11–0.21) (Fig. 4b and Additional file 7: Table S3). Intron retention, a characteristic of cancer transcriptomes [14], was also observed in 31 genes across the various samples (Fig. 4b). Of interest, 15 out of 18 of the B-ALL samples had a retained intron in *ZCCHC7* involving intron 2 (Fig. 5a), which is in a region known for breakpoints in pediatric B-ALL [15]. Furthermore, a novel cryptic gene fusion was identified in one of T-ALL samples involving *SPTAN1* and *ABL1* (Fig. 5b).

In addition to the StVs, 170 SNVs were called. Almost half of SNVs (47%) were localized to the untranslated regions (UTR), with missense and synonymous mutations being the second (26%) and third (17%) most common SNVs, respectively (Fig. 4c). SNVs causing frameshift mutations were found in 5% of variants, while nonsense mutations were found in 1% variants. 4% of SNVs were detected in exon:intron splice junctions, and the majority of the coding variants were located within *NOTCH1* (Fig. 4d).

## Discussion

The heterogeneity of clinically relevant, low VAF genetic variation in healthy individuals and leukemia patients is far more diverse than previously appreciated and not readily detectable by standard diagnostics or even “deep sequencing” without UMIs. The clinical significance of this variation is only beginning to be understood, but as the repertoire of precision therapeutics in cancer expands, the optimization of risk prognostication and therapeutic selection requires precise quantification multiple cancer-related mutations in DNA and RNA simultaneously at frequencies as low as 0.0001, two orders of magnitude below the error rate of current NGS platforms.

This diversity requires a “toolbox” of sensitive and specific molecular and computational strategies for any leukemia, such as we have outlined here. The implementation of a molecule-specific, random indexing to enables the computational removal of stochastic sequencing and PCR errors to accurately call mutations at very low levels. These strategies can be used for clonal profiling and disease surveillance across a wide variety of targets simultaneously. The limit of detection established in this report are equivalent to the current flow-based methods and, as demonstrated, ECS is capable of overcoming many limitations of traditional NGS assays [14]. For instance, while a clinical molecular MRD assay does exist for *FLT3-ITD* (Lin et al., 2015), it is only a single mutation assay and is not capable of detecting clinically relevant co-occurring mutations. Furthermore, the detection of ITDs > 80 bp has historically been quite difficult with anything other than whole genome sequencing, including hybridization methods [16].

Another interesting observation is the various types of hematopoietic clones apparent in a single individual at different points in time during therapy. We intended to characterize the disappearance of AML-associated mutations from diagnosis to EOI and how many of these clonal mutations reappeared in those that relapsed. However, we find various classes of mutations at each time point. In addition to the expected loss of AML-related clonal mutations, we see mutations that are extremely stable at each time point, apparently unaffected by cytotoxic chemotherapy, but unrelated to the patient’s leukemia.

The number of clonal mutations gained after induction therapy is quite similar to the number of clones that disappear after induction chemotherapy, resulting in a roughly stable absolute number of hematopoietic clones, focusing relevance on the nature of the mutation rather than the abundance. Finally, there are some clones that actually appear to increase after induction therapy, suggesting they have a growth advantage, which is akin to prior reports of pre-existing *TP53*-mutated hematopoietic clones that expand following chemotherapy for a primary malignancy, but sometimes acquire additional mutations that result in transformation to therapy-related AML [17]. Of these, mutations in *IKZF1* were the most common to demonstrate an increased VAF at EOI for several of the subjects, and several of the subjects had a deletion as similar described by de Rooij et al. [18]. These observations must be weighed against the small sample size and the fact that this sequencing panel was limited in breadth.

## Conclusion

ECS coupled with an AMP approach enables the detection of complex low allelic variants. This technique has the potential to further advance our understanding of MRD and personalized medicine. Further studies utilizing single cell DNA or RNA sequencing that can quantify allele-specific expression would provide important additional insights for mutation co-localization and associated impacts on gene expression and cellular phenotype. While future ECS-based studies could provide better resolution than current flow-based MRD assays, correlating identified mutations with true disease, risk stratification and therapeutic selection will require much larger, well phenotype studies.

## Supplementary information

**Additional file 1: Figure S1.** Bioinformatics utilities for variant detection. The workflow described consists of 3 major areas: capture technique, alignment method, and variant detection. In this report we focused on AMP and amplicon based technologies. Two different alignment methods were used: force reference alignment and de novo assembly. Multiple methods were used for variant detection including freebayes, LoFreq, and ARBA. The results from the various algorithms are ultimately merged and displayed in our custom graphical interface.

**Additional file 2: Figure S2.** Schema of error-corrected sequencing molecules. Library preparation for ECS includes the addition of a molecular barcode (dark grey box) that enables identification of molecular bins. Each subject is given a sample index (green box) during library preparation which allows multiple samples to be pooled during sequencing. Each molecule contains a universal primer site (purple box) and a random adaptor ligation (yellow box).

**Additional file 3: Figure S3.** Graphical representation of a CNV loss in CBL. Each of the dots represents a gene specific primer (GSP). Deviation from 2 copies are represent below the line (loss) or above (gain).

**Additional file 4: Figure S4.** RNA-ECS is accurate to single transcripts without normalization. (A and B) Technical replicates from umbilical cord blood and a pediatric AML remission bone marrow aspirate. Absolute numbers of transcripts for all genes with fewer than 100 called copies are plotted (genes with > 100 copies were not included on the plot to highlight limit of detection) showing strong technical replication. (C) Transcript counts spanning two orders of magnitude were validated via ddPCR, showing a strong concordance.

**Additional file 5: Table S1.** Summary of demographics for the pediatric leukemia samples.

**Additional file 6: Table S2.** Genes targeted on the DNA and RNA panels.

**Additional file 7: Table S3.** Structural variants identified via RNA-ECS in primary diagnostic samples.

## Abbreviations

ALL: Acute lymphoblastic leukemia
AML: Acute myelogenous leukemia
AMP: Anchored multiplexed PCR
APL: Acute promyelocytic leukemia
CNV: Copy number variation
COG: Children’s Oncology Group
ECS: Error-corrected sequencing
FISH: Fluorescence in situ hybridization
GSPs: Gene-specific primers
ITD: Internal tandem duplication
LAIP: Leukemia-associated immunophenotype
MPFC: Multi-parameter flow cytometry
MRD: Minimal residual disease
NGS: Next generation sequencing
SNVs: Single nucleotide variants
StVs: Structural variants
TARGET: Therapeutically Applicable Research to Generate Effective Treatments
UMI: Unique molecular index
UTR: Untranslated regions
VAF: Variant allele frequency
