# Supplementary figures and images for: Error-corrected sequencing strategies enable comprehensive detection of leukemic mutations relevant for diagnosis and minimal residual disease monitoring
Source: BMC Med Genomics. 2020 Mar 4;13:32. doi: 10.1186/s12920-020-0671-8 (PMC7057603; doi:10.1186/s12920-020-0671-8)

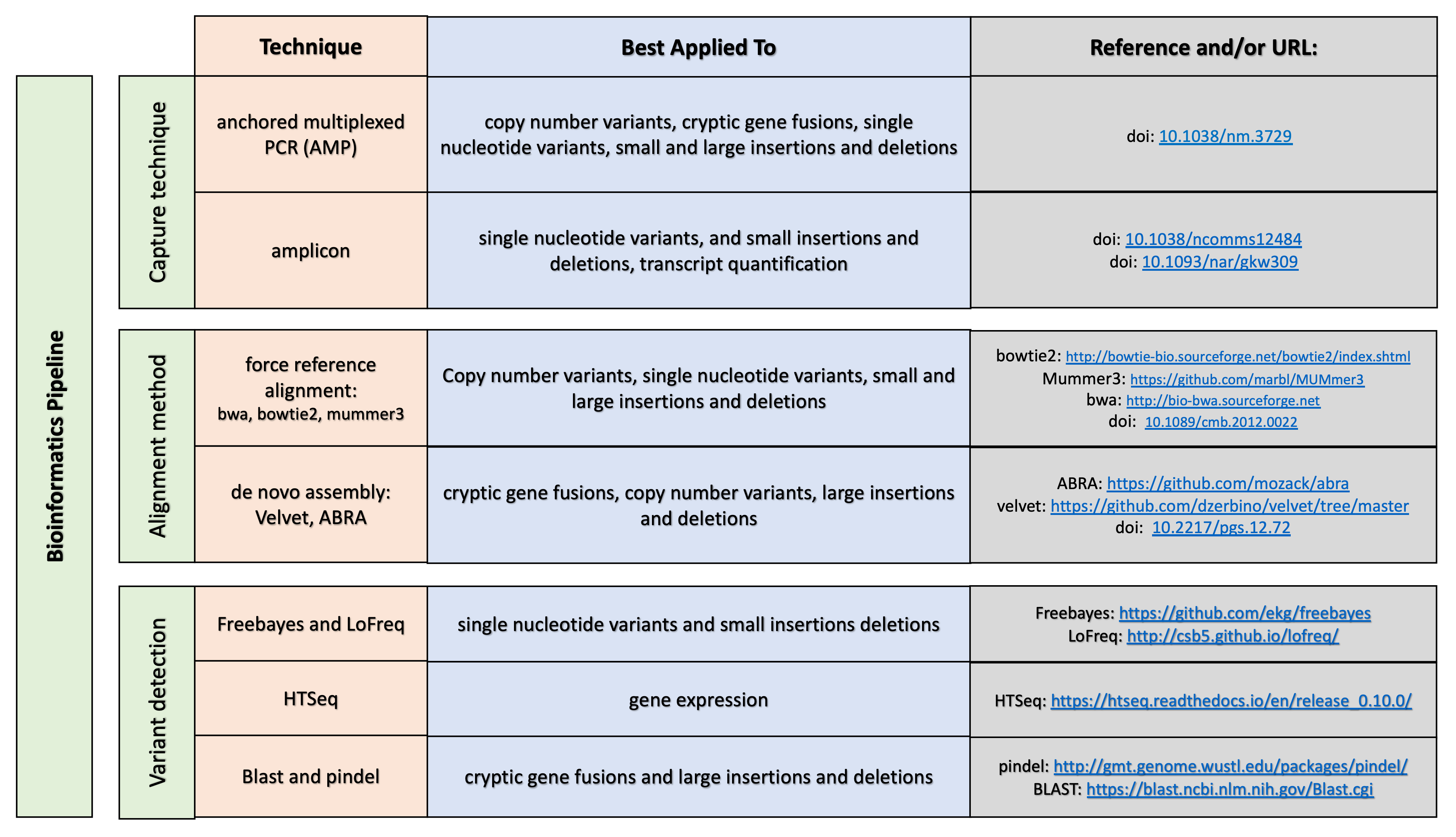

Supplement: Supplementary file 1 — Additional file 1: Figure S1. Bioinformatics utilities for variant detection. The workflow described consists of 3 major areas: capture technique, alignment method, and variant detection. In this report we focused on AMP and amplicon based technologies. Two different alignment methods were used: force reference alignment and de novo assembly. Multiple methods were used for variant detection including freebayes, LoFreq, and ARBA. The results from the various algorithms are ultimately merged and displayed in our custom graphical interface. [file 12920_2020_671_MOESM1_ESM.jpg]

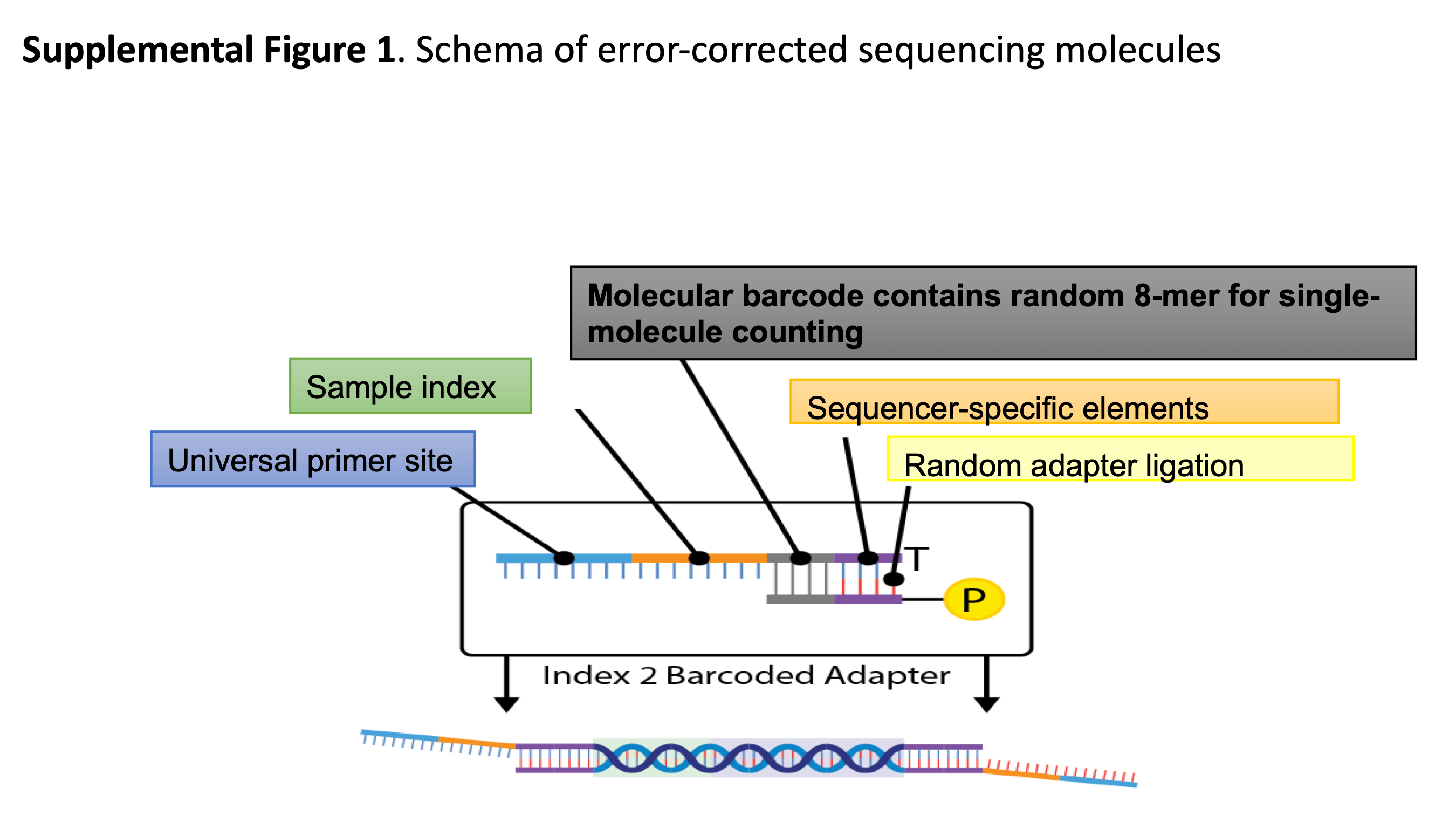

Supplement: Supplementary file 2 — Additional file 2: Figure S2. Schema of error-corrected sequencing molecules. Library preparation for ECS includes the addition of a molecular barcode (dark grey box) that enables identification of molecular bins. Each subject is given a sample index (green box) during library preparation which allows multiple samples to be pooled during sequencing. Each molecule contains a universal primer site (purple box) and a random adaptor ligation (yellow box). [file 12920_2020_671_MOESM2_ESM.jpg]

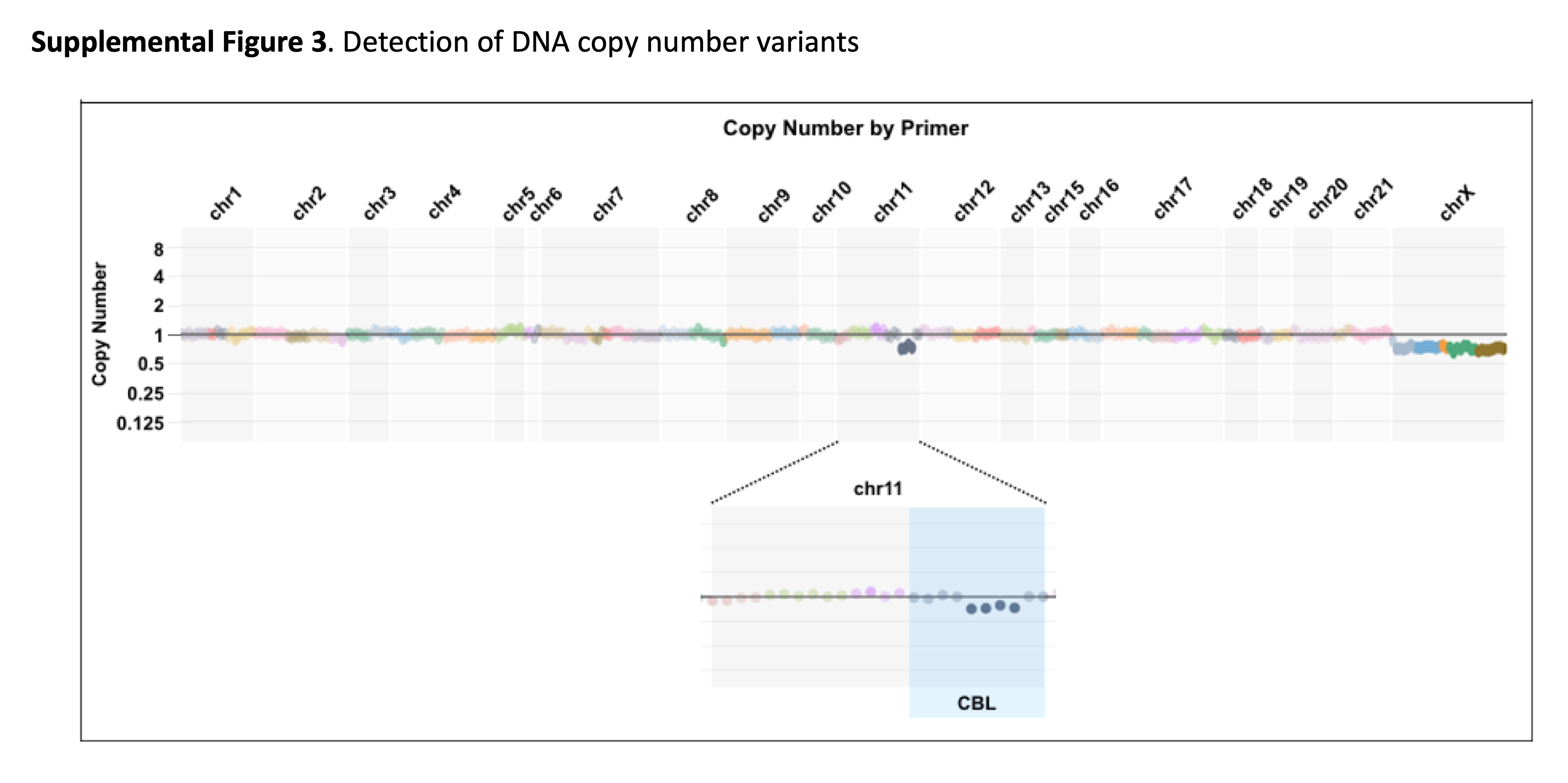

Supplement: Supplementary file 3 — Additional file 3: Figure S3. Graphical representation of a CNV loss in CBL. Each of the dots represents a gene specific primer (GSP). Deviation from 2 copies are represent below the line (loss) or above (gain). [file 12920_2020_671_MOESM3_ESM.jpg]

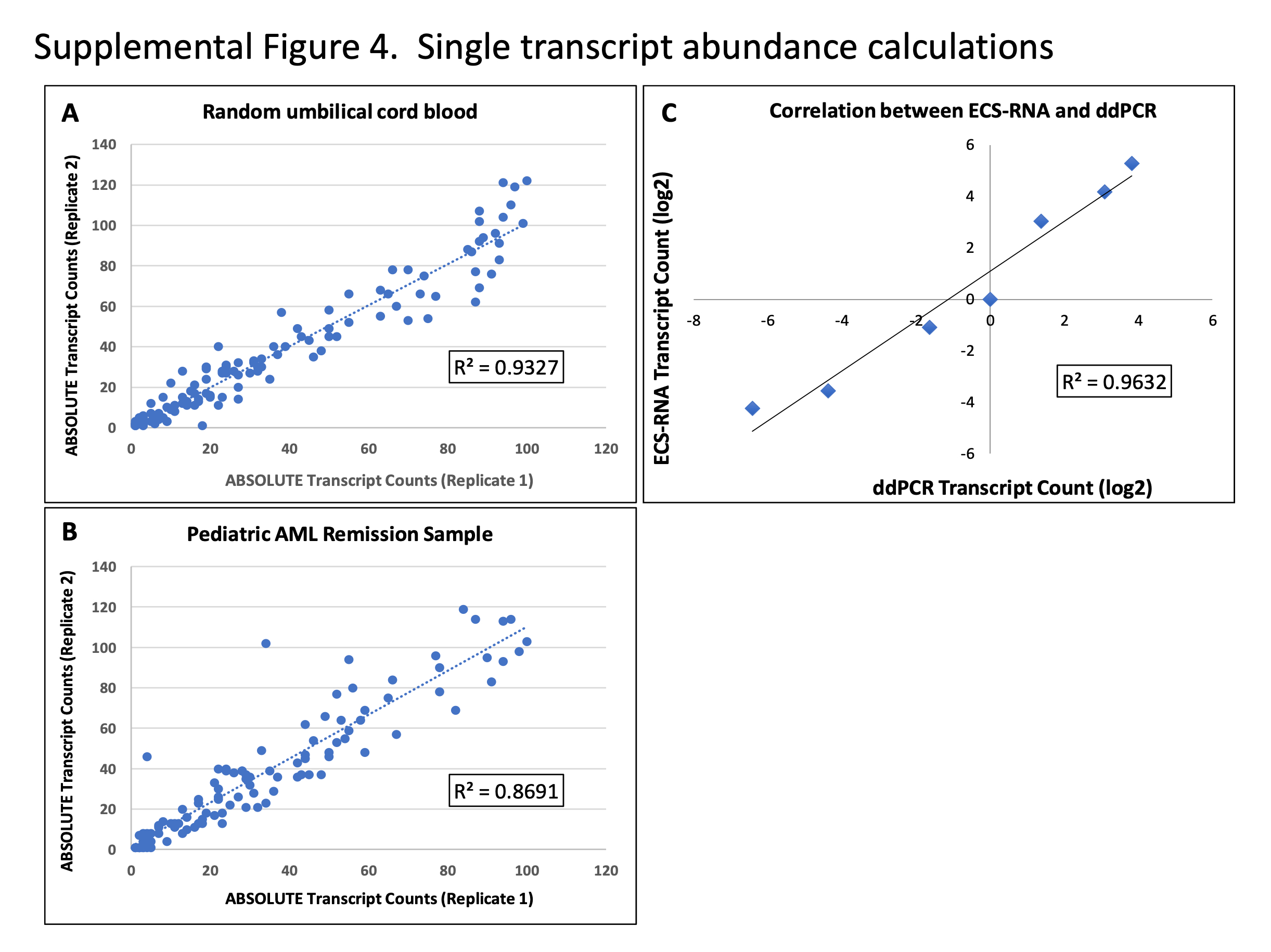

Supplement: Supplementary file 4 — Additional file 4: Figure S4. RNA-ECS is accurate to single transcripts without normalization. (A and B) Technical replicates from umbilical cord blood and a pediatric AML remission bone marrow aspirate. Absolute numbers of transcripts for all genes with fewer than 100 called copies are plotted (genes with > 100 copies were not included on the plot to highlight limit of detection) showing strong technical replication. (C) Transcript counts spanning two orders of magnitude were validated via ddPCR, showing a strong concordance. [file 12920_2020_671_MOESM4_ESM.jpg]
